# Swimming turn performance: the distinguishing factor in 1500 m world championship freestyle races?

**DOI:** 10.1186/s13104-021-05665-x

**Published:** 2021-06-30

**Authors:** Marek Polach, Dan Thiel, Jan Kreník, Dennis-Peter Born

**Affiliations:** 1 Faculty of Physical Culture, Department of Social Sciences in Kinanthropology, Palacký University Olomouc, Olomouc, Czech Republic; 2 Faculty of Physical Education and Sport, Department of Physiology and Biochemistry, Charles University Prague, Prague, Czech Republic; 3 Department for Competitive Swimming, Czech Swimming Federation, Praha, Czech Republic; 4 Department for Elite Sport, Swiss Federal Institute of Sport Magglingen, Magglingen, Switzerland; 5 Department for High-Performance Sports, Swiss Swimming Federation, Berne, Switzerland

**Keywords:** Elite athletes, Front crawl, Performance analysis, Start

## Abstract

**Objective:**

Turn sections represent the second largest part of total race time in 1500 m freestyle races and may substantially affect race results. Therefore, the aim of the study was to investigate individual race strategies and compare the effect of start, swim, and turn performances between short-course and long-course races. Video footages were collected from all 16 male finalists at the 2018 short and 2019 long-course World swimming championships (age 23.06 ± 2.3 years, FINA points 941 ± 42) for subsequently analysis of start, turn, and swim performance.

**Results:**

The larger number of turns in short-course races resulted in significantly faster race times (*p* = 0.004), but slower mean turn times compared to long-course races (*p* < 0.001). Total race velocity closely correlated with swim and turn but not start section velocity in short- (*r* ≥ 0.80, *p* ≤ 0.017) and long-course races (*r* ≥ 0.83, *p* ≤ 0.011). Analysis of individual race strategies showed that turn performance affected race results in 6 (75%) and 3 (37.5%) of the 8 world-best 1500 m swimmers in short-course and long-course races, respectively. Medal standing was improved for 1st, 3rd, and 4th ranked short- as well as 1st and 2nd ranked long-course finalist. Coaches, athletes, and performance analysts may carefully consider the importance of turn performance additionally to free-swimming skills.

## Introduction

Performance analysis has become routine procedure in high-performance sports, in order to evaluate the training process, discover potentials, and investigate key performance indicators [1, 2]. As pool swimming is affected by little environmental factors, swimming performance can be assessed based on real race scenarios with no equipment interfering with the swimmer´s movement pattern [3, 4]. Performance analysts commonly divide swim races into several sections. The start section includes the block phase, flight phase, underwater phase, and transition to full-stroke swimming [5, 6]. Contribution of start performance in 50 m events was 26.1% [7] but continuously decreased for 100 m and 200 m races [8, 9] and may be of minor importance for 1500 m freestyle [10]. Here, turns that are used for directional change and to reaccelerate the swimmer by pushing of the pool wall at the beginning of each lap may substantially affect the race result [11]. The turn sections are commonly analyzed from 5 m before wall contact until resurfacing after the underwater phase, which varies in its length depending on the race distance [12, 13]. Distance between the start and turns determine swim section [3, 8, 14].

While parameters related to free-swimming have been extensively investigated [1, 15–17], turns represent 19.69 ± 0.24% [8] and 36.87 ± 0.61% [18] of total race time in 100 m and 1500 m long-course freestyle races, respectively. Significant performance variations were attributed to turn parameters, i.e. 5 m in (time before wall contact), breakout time, breakout distance, 15 m out (time after wall contact) [8, 10, 18]. However, in these particular studies, turn sections were based on FINA rules that allow an underwater phase up to the 15 m mark [19]. As mean breakout distance was 5.48 ± 0.87 m in these 1500 m races [18], free-swimming skills may have large affected turn performance. Therefore, previous studies suggested to isolate the turn and swim sections [12, 13] with particular attention to the last 5 m before and initial 5 m after wall contact [20, 21].

In long-course races (50 m pool length), the effect of turn performance on race results increased with race distance, hence number of turns involved [8–10, 18]. As the number of turns is twice as high in 1500 m short-course races (25 m pool length), turn performance may show an even larger effect on race results. While analysis of world-class athletes provides unique insights into human’s highest possible performance, such analyses are naturally limited by a small number of subjects [22, 23]. Hence, performance at World championship level should be investigated based on individualized responses and case reports in addition to the assessment of mean values [24–26]. Therefore, the aim of the study was to investigate individual race strategies of World championship finalists and compare the effect of start, swim, and turn performances on results of 1500 m freestyle events between short-course and long-course races.

## Main text

### Materials and methods

#### Participants

Video footages were collected from 8 male finalists of the 1500 m freestyle events at the Hangzhou 2018 (age 22.8 ± 2.4 years, FINA points 953 ± 27) (short-course) and Gwangju 2019 (age 23.3 ± 2.2 years, FINA points 951 ± 23) (long-course) World championships for subsequent analyses of start, turn, and swim sections. All participants of the FINA World swimming championships provided written informed consent to the organizer that all video material collected during competition can be used for television broadcasting and race analyses by the participating nations. All data were anonymized before the analysis. The study was approved by the Ethical committee of the Palacký University of Olomouc (Registration-Number: 77/2020) and in accordance with the Declaration of Helsinki.

#### Data collection

For the short-course World championships, a camera (Canon XA35, Canon Inc., Tokio, Japan) was positioned on top of the stands 30 m above water level and about 100 m from the side of the pool. The camera was placed perpendicular to the direction of swimming and exactly in the middle of the pool (12.5 m apart from both pool ends). The camera zoom was set before the races to assure clear vision across the entire pool. To ensure same conditions for the long-course World championships (50 m pool length), two cameras of the same type used before, were positioned 12.5 m apart from both ends of the pool. Cameras were synchronized via wireless LAN connection and recorded half to the pool each. Video footages were recorded as mp4 format with 50 frames-per-seconds and an image qualify of 1920 × 1080 full HD.

#### Data analysis

Split times for start, swim, and turn sections were analysed using Dartfish (Team pro Data 9, Dartfish, Fribourg, Switzerland). The light flash of the timing system that was synchronized to the starting signal was used to synchronize video footages for the race analysis. Race results were obtained from the official electronical timing system (Omega Timing, Biel/Bienne, Switzerland).

Based on mean breakout distances of 10.69 ± 1.18 m and 10.11 ± 1.25 m for short- and long-course races, respectively, start section was determined by the time from the starting signal until top of the swimmer’s head passed the 10 m mark visible on the lane ropes. Based on previously reported breakout distances between 4.64 ± 0.23 m and 5.48 ± 0.87 m in 1500 m freestyle races [18, 27], turn sections were analysed from 5 m before until 5 m after wall contact. Swimmer’s head passing the 5 m mark (short- and long-course), 20 m mark (short-course), and 45 m mark (long-course) visible on the lane ropes were used as reference points [28]. Race sections beyond start and turn section determined the swim section. Accuracy of all markers were checked using a measuring tape before the race. To compare a potential fatigue effect between short- and long-course races, mean turn times were compared across the 1st, 2nd and 3rd 500 m section of each event.

#### Statistical analysis

The data are presented in mean ± standard deviation (SD). Normality was verified with Shapiro-Wilks test and alpha-level of 0.05 indicated statistical significance. Spearman’s correlation coefficient was applied, to assess relationship between mean velocities and ranking in all race sections. For Spearman’s correlation, a critical value of 0.643 with an alpha level of 0.05 was reported to a data sample of eight participants [29]. Analysis of variance (ANOVA) was used with section times as the dependent variable and pool length (short-course vs. long-course) and race section (start vs. swim vs. turn) as categorical factors with Tukey´s *post-hoc* test. A one-way ANOVA was used to compare total race time between short- and long-course races. To investigate accuracy, 50% of all races were analyzed by another experienced race analysts. Inter-rater reliability was assess using Intraclass-correlation coefficient (ICC) between the repeated measures and showed an ICC of 0.988–0.989 and 0.991–0.992 for short-course and long-course World championships, respectively. Statistical analysis was performed using the STATISTICA software version 13.4.0.14. (StatSoft Inc., Tulsa, USA).

## Results

The times for start, swim, and turn sections are presented in Table 1 (mean ± standard deviation) for short-course and long-course 1500 m races. Total race time was significantly faster for short-course (865.09 ± 12.90) compared to long-course (889.57 ± 13.98; *p* = 0.004). Additionally, in short-course, swimmers spent significantly less time in the swim (64.52% vs. 83.04%; *p* < 0.001) and more time in the turn sections (35.04% vs. 16.53%; *p* < 0.001) compared to long-course, respectively. However, mean turn times were significantly faster for long-course compared to short-course (5.07 ± 0.18 vs. 5.14 ± 0.10 s; *p* < 0.001).

**Table 1:** Descriptive data with mean ± standard deviation (SD). Section times were compared between pool lengths and race section using a mixed-design analysis of variance (ANOVA)

	Short-course World championships *25 m pool length – Hangzhou 2018*	Long-course World championships *50 m pool length – Gwangju 2019*		ANOVA	
	Mean ± SD 95% CI lower bound–upper bound	Mean ± SD 95% CI lower bound–upper bound		*F*-value	*p*-value
Total start section time [s]	3.77 ± 0.16 3.66–3.88	3.77 ± 0.17 3.65–3.89	a)	18	< 0.001
Total swim section time [s]	558.12 ± 9.47 * 551.56–564.68	738.68 ± 9.47 * 732.35–745.01	b)	38859	< 0.001
Total turn section time [s]	303.14 ± 5.71 * 299.18–307.10	147.12 ± 5.71 * 143.58–150.66	c)	2569	< 0.001
Mean start section time [s]	3.77 ± 0.16 3.66–3.88	3.77 ± 0.17 3.65–3.89	a)	18976	< 0.001
Mean swim section time [s]	9.30 ± 0.16 * 9.19–9.41	24.62 ± 0.33 * 24.39–24.85	b)	46166	< 0.001
Mean turn section time [s]	5.14 ± 0.10 * 5.07–5.21	5.07 ± 0.18 * 4.95–5.19	c)	19079	< 0.001

a) Main effect: pool length (short-course vs. long-course)

b) Main effect: race section (start vs. turn vs. swim)

c) Interaction effect: pool length x type of race section

^*^significant difference between short- and long-course races (*p* < 0.05)

Analysis of variance (ANOVA) revealed that mean turn times of the first 500 m section were not different between short-course and long-course (*p* = 0.381). Thereafter, turn times became significantly slower for the short-course (*p* < 0.001) but remained stable for the long-course races (*p* = 0.732).

Correlation analysis revealed close correlations between the final ranking with swim and turn section ranking in both, short- (*r* = 0.074, *p* = 0.037; *r* = 0.81, *p* = 0.015) and long-course (*r* = 0.93, *p* < 0.001; *r* = 0.93, *p* < 0.001). Additionally, total race velocity correlated with swim and turn section velocities in short- (*r* = 0.80, *p* = 0.017; *r* = 0.81, *p* = 0.015) and long-course (*r* = 0.98, *p* < 0.001; *r* = 0.83, *p* = 0.011). Start performance was not related to final ranking or total race velocity (Table 2).

**Table 2:** Spearman’s correlation coefficient between final ranking and section rankings as well as total race velocity and section velocities

Final ranking	Section ranking			Total race velocity	Section velocity [m.s^−1^]		
	Start	Swim	Turn		Start	Swim	Turn
Short-course world championships 25 m pool length –Hangzhou 2018							
1. Place	5	2	1.^a^	1.77	2.62	1.64	2.00^a^
2. Place	8	1	4.^b^	1.76	2.45	1.65	1.96^b^
3. Place	3	6	2.^a^	1.75	2.75	1.61	1.99^a^
4. Place	4	5	3.^a^	1.74	2.81	1.61	1.97^a^
5. Place	6	4	6.^b^	1.74	2.67	1.63	1.92^b^
6. Place	1	3	8.^b^	1.73	2.59	1.63	1.90^b^
7. Place	7	7	5	1.71	2.58	1.59	1.93
8. Place	2	8	7	1.68	2.78	1.56	1.90
* r-*value	− 0.36	0.74	0.81	*r*-value	− 0.20	0.80	0.81
* p-*value	0.385	0.037	0.015	*p*-value	0.629	0.017	0.015
Long-course world championships 50 m pool length – Gwangju 2019							
1. Place	2	2	1.^a^	1.71	2.76	1.64	2.04^a^
2. Place	4	3	2.^a^	1.71	2.70	1.64	2.03^a^
3. Place	7	1	4.^b^	1.71	2.53	1.65	1.99^b^
4. Place	3	4	5	1.70	2.72	1.63	1.99
5. Place	1	5	3	1.69	2.86	1.62	2.03
6. Place	6	6	6	1.68	2.55	1.62	1.96
7. Place	8	7	7	1.66	2.49	1.61	1.90
8. Place	5	8	8	1.63	2.67	1.58	1.84
* r-*value	0.43	0.93	0.93	*r*-value	0.32	0.98	0.83
* p-*value	0.289	< 0.001	< 0.001	*p*-value	0.444	< 0.001	0.011

^a^increased final ranking due to turn performance

^b^decreased final ranking due to turn performance

**Fig. 1:**
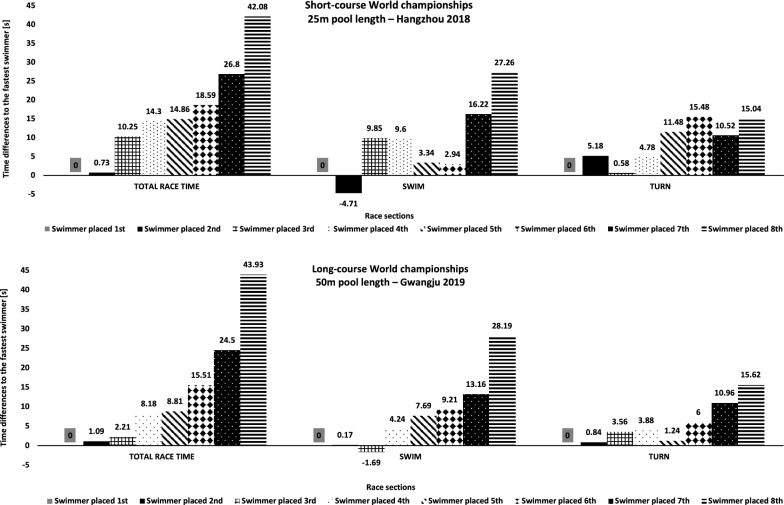
Individual differences to the fastest swimmer for total race time, swim, and turn section at the short-course and long-course world championships

Figure 1 illustrates the individual response of the swimmer’s swim and turn performance on final ranking. Turn performance affected final ranking in 6 (75%) and in 3 (37.5%) out of 8 short- and long-course World championship finalists, respectively. In particular, for the highest ranked swimmers, turn performance improved medal standing, i.e. 1st, 3rd, and 4th ranked short-course finalist as well as 1st and 2nd ranked long-course finalist. For instance, in short-course races, the swimmer ranked 2nd (545.35 s) swam 4.71 s (0.86%) faster than the winner of the race (550.06 s) but lost 5.18 s (1.75%) in the turn sections. Moreover, swimmer ranked 3rd only showed the 6th fastest swimming time. However, this swimmer gained up to 14.9 s (5.24%) due to the turns and outperformed the 4th, 5th, and 6th ranked swimmers with second fastest total turn time. In long-course races, swimmer ranked 3rd swam 1.69 s (0.23%) and 1.86 s (0.26%) faster than the 1st and 2nd ranked swimmer. However, final ranking was determined by faster turn times for 1st (3.56 s; 2.51%) and 2nd (2.72 s; 1.92%) compared to 3rd ranked swimmers.

## Discussion

The present study showed importance of turn performance in 1500 m freestyle short- and long-course races. The push-off from the pool wall and subsequent underwater phase with application of undulating kicking accelerates swimmer above free-swimming speed [30]. Swimmers aim to lengthen the underwater phase, as drag forces are lower under water than at its surface [31]. However, excess breath holding increases anaerobic demand and may interfere with swimmers free-swimming abilities [32]. Therefore, with increasing race distance, swimmers successively reduce length of underwater phase down to 4.64 ± 0.23 m in the 1500 m freestyle [27]. While long-distance swimmers apply rather slow and energy-saving leg kicking [33], push-off from the wall and undulating kicking during the underwater phase place a high demand on leg muscles [11]. Future research needs to investigate whether length of the underwater phase and conditioning of leg muscles may provide potential for future performance developments in long-distance swimmers.

The present and previous studies showed significantly faster race times in short- compared to long-course races [34] probably due to twice the number of turns involved, hence repeated velocity gains from wall push-off [30, 35]. However, mean turn performance was slower in short-course races. A detailed analysis revealed equal turn times in the first 500 m sections of short- and long-course races. However, with the remaining two 500 m sections, turn times became slower in short- but not long-course races. The high demand for leg muscles during push off from the wall [11, 33] and repeated breath holding during the underwater phase [32] may have resulted in a fatigue effect during short-course races. However, swimmers accelerate beyond free-swimming speed with the push-off after the turn and swimming performance should basically benefit from the larger resistance provided by the pool wall assuming an adequate conditioning for leg muscles [36]. Therefore, swimmers may have applied a more careful pacing strategy and more energy conserving turns due to higher number of turns available in short-course races. Although metabolic energy supply cannot be decoupled between the turn and swim section, based on faster turn times on long-course races, short-course races may have not yet found its limit [37]. The question arises whether the in average 0.07 s faster turn times seen in long-course races could be applied in 25 m pool competition, hence short-course races. With 30 additional turns this would add up to a performance gain of 2.03 s and beat the current world record [38] by 0.95 and 0.22 s for the 1^st^ and 2^nd^ ranked swimmer of the recent 2018 World short-course swimming championships. However, multiple variables interact in a 1500 m freestyle race and the present hypothesis is yet to be evaluated by future studies.

## Conclusion

The present study showed that turn performance could be the distinguishing factor in World-championship 1500 m freestyle races. Turn performance affected final ranking in 6 (short-course) and in 3 (long-course) out of 8 World championships finalists. Coaches, athletes, and performance analysts should carefully consider the importance of turn performance in addition to free-swimming skills.

## Limitations

Analyses in top-elite athletes, i.e. the eight world best 1500 m swimmers in the short- and long-course championships, naturally come with a low number of participants. Although the current results of such a low number of world-class swimmers cannot be translated to a general population of competitive swimmers, such analyses provide unique insights into individual race strategies. Marginal differences should not be neglected as they may provide the distinguishing factor for final ranking. Therefore, further studies need to verify these findings based on data collections across finalists of multiple World championships. Additionally, individualized distances measurements of underwater phase and breakout distances after the turn would allow further insights and a detailed analysis of turn strategies [12].

## Data Availability

All data are available on request from the corresponding author Marek Polach.
